# The Potential Benefit of Hydroxychloroquine in Chronic Placental Inflammation of Unknown Etiology Associated with Adverse Pregnancy Outcomes

**DOI:** 10.3390/healthcare10010168

**Published:** 2022-01-17

**Authors:** Alexandra Bouariu, Nicolae Gică, Anca Marina Ciobanu, Ana Maria Scutelnicu, Mihaela Roxana Popescu, Anca Maria Panaitescu

**Affiliations:** 1 Department of Obstetrics and Gynecology Filantropia, Clinical Hospital Bucharest, 011171 Bucharest, Romania; Alexandra.bouariu@yahoo.com (A.B.); gica.nicolae@umfcd.ro (N.G.); ciobanu.ancamarina@gmail.com (A.M.C.); ana.scutelnicu@yahoo.com (A.M.S.); 2 Department of Obstetrics and Gynecology, Carol Davila University of Medicine and Pharmacy, 050474 Bucharest, Romania; 3 Department of Cardiology, Elias University Hospital Bucharest, 011461 Bucharest, Romania

**Keywords:** hydroxychloroquine, chronic placental inflammation, chronic villitis, chronic intervillositis of unknown origin, chronic histiocytic intervillositis

## Abstract

The placenta is the site of connection between maternal and fetal circulation, and the liaison is established early in pregnancy. A large variety of pregnancy complications such as preterm birth, fetal growth restriction, or pregnancy loss have placental expression and can be accompanied in some cases of acute or chronic identifiable placental inflamatory lesions. Chronic placental inflammatory (CPI) lesions include chronic villitis of unknow etiology (CVUE), chronic intervillositis of unknown etiology, CIUE (also described as chronic histiocytic intervillositis, CHI), and chronic deciduits. Hydroxychloroquine (HCQ) has been prescribed with good results during pregnancy to prevent adverse perinatal outcomes in maternal autoimmune conditions. Its success has paved the way to its use in CPI as CIUE/CHI; however, to date, there are no prospective, informatively designed, controlled studies on its value in these setting. This review aims to explore the potential role of HCQ in CPI of unknown etiology. Ideally, properly designed, probably multicentric studies should be undertaken to fully understand HCQ’s role for prevention of adverse pregnancy outcomes after a chronic placental inflammation.

## 1. Introduction

Considered central to chronic disease development [1], placental phenotype arrangement is thought to determine chronic adult-onset disease. Unbalanced maternal nutrition, periods of chronic hypoxia or increased levels of glucocorticoids or thyroid hormones determine fetal structural alterations such as reduced blood vessel diameter [2], low arterial elastin [3], reduced numbers of nephrons in the kidney [4], reduced number of beta cells in the pancreas [5], and changes in brain structure and function [6] that increase the vulnerability for heart disease, stroke, obesity, and diabetes later in adult life.

**Figure 1:**
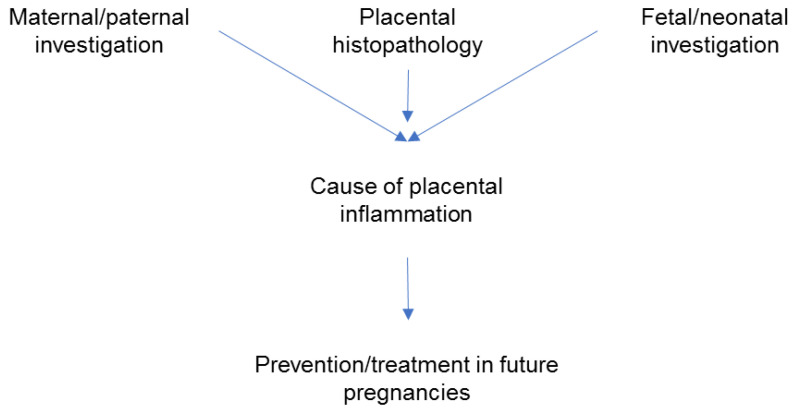
Steps in the clinical management of adverse pregnancy outcomes associated with placental inflammation; the focus is on prevention in subsequent pregnancies.

The placenta is the site of connection between maternal and fetal circulation and the liaison is established early in pregnancy, when placentation occurs. Therefore, a large variety of pregnancy complications have placental expression. Inflammatory placental conditions with acute or chronic onset have specific immunological mechanisms and carry a significant short- and long-term response in fetal development with an increased recurrence rate for subsequent pregnancies. Acute placental inflammation, as seen on microscopical preparations, is associated to chorioamnionitis [7]. The origin of chorioamnionitis includes amniotic fluid infection, intrauterine infection, or ascending infection [8]. Bacteria are rarely identified at term [9], but more frequently identified in preterm deliveries when acute inflammation of the placenta and clinical signs of chorioamnionitis are present [10]. Forces of labor themselves [11] and maternal comorbidities (obesity) [12] induce inflammation that may be reflected in the placenta. Chronic placental inflammation (CPI) lesions involve specific cells, such as lymphocytes and histiocytes and have a particular location in the placenta [13]. They may be associated with autoimmune disorders or persistent infection, or may be of unknown etiology. Chronic inflammation decreases the healthy tissue involved in uteroplacental circulation and is linked to severe obstetric complications such as fetal growth restriction (FGR), preterm birth (PTB), and pregnancy loss [14]. Chronic inflammation of the placenta can be suspected during pregnancy if complications such as recurrent miscarriage, stillbirth, or FGR develop, but confirmation is only made after delivery in a histopathological exam [14]. The clinical approach is to look for a cause of the placental inflammation by combining information provided by the pathology exam and the investigations performed in the mother, father, fetus, or neonate. Discovering a cause is important for subsequent pregnancies management (Figure 1) since some forms of CPI are recurrent [14].

A better understanding of the chronic inflammatory process in the placenta is needed in view of possible methods of treatment, prevention, and better pregnancy outcomes. Various drugs have been tried with mixed success rates to improve outcomes in subsequent pregnancies after demonstration of CPI in histological specimens: steroids, aspirin, low molecular weight heparin, and intravenous immunoglobulins [15,16,17,18].

The antimalarial agent hydroxychloroquine (HCQ) has emerged as a safe drug to be used during pregnancy for preventing adverse outcomes in mothers with autoimmune conditions [19,20,21,22,23,24,25,26,27], where its beneficial effect is considered to outweigh the potential risks to the fetus. This has encouraged taking it into consideration for prevention of recurrent CPI lesions [27]. However, caution should be exerted whenever a drug is used with new indications without properly conducted research. Hydroxychloroquine in particular was the drug involved in the so called “hype-based medicine” for treatment of COVID-19 infections in 2020 [28].

The aim of this review was to explore the literature related to the potential role of hydroxychloroquine in chronic placental inflammation of unknown etiology. We also aimed to increase awareness of the association of pregnancy adverse outcomes with chronic placental inflammation. In addition, we want to underline that the placenta should always be sent for analysis in cases of pregnancy complications and that, ideally, all obstetric facilities should partner with a specialized pathologist with experience in placenta histopathology.

## 2. Types of Chronic Placental Inflammation

The Amsterdam classification system defines four major patterns of placental injury: maternal vascular malperfusion, fetal vascular malperfusion, acute chorioamnionitis, and villitis of unknown etiology [8]. It recognizes other inflammatory lesions reported to be associated with villitis, such as eosinophilic/T-cell vasculitis, chronic intervillositis, and chronic deciduitis. Eosinophilic/T-cell vasculitis is characterized by the presence of T lymphocytes and eosinophils in the chorionic plate vessels [29]. Chronic intervillositis is characterized by infiltration of the intervillous space by histiocytes and is particularly associated with adverse pregnancy outcomes and can be recurrent. It can be found as a predominant feature or in association with chronic villitis [15,30,31,32]. Recently, a proposition for standardized criteria for uniformity in its diagnosis has been proposed [33]. Chronic deciduitis is defined by chronic inflammation, and the presence of lymphocytes and plasma cells in the basal plate [8].

The histological analyses may reveal areas of inflammatory cells such as lymphocytes, histiocytes, and plasmocytes aggregated within the placenta sometimes with a particular specific location and specific genotypes and phenotypes [34,35,36]. Maternal infections such as COVID-19 and cytomegalovirus infection can cause chronic placental inflammation with possible consequences to the fetus; however, unfortunately, a specific etiology is not identifiable in most of the placental inflammation cases [34]. Table 1 summarizes the classification of CPI.

It is considered that CPI may be related to failure of the maternal tolerance to fetal antigens and with maternal immune system activation; however, the complete pathogenesis is not completely understood [34].

In chronic villitis of unknown etiology (CVUE) maternal immune rejection of a semi allogeneic placenta is thought to be a mechanism [16]. In patients with recurrent pregnancy loss, the prevalence is reaching 8% and the recurrence in subsequent pregnancies ranges from 18% to 100% [31]. Chronic villitis of unknown etiology involves a large infiltration of mainly placental terminal villi by lymphocytes and histiocytes, as well as, less commonly, plasma cells. The cell aggregation is characterized by destruction of capillaries resulting in poor uteroplacental circulation. A field that requires further investigation and studies is the treatment of subsequent pregnancies after a pregnancy loss that demonstrates the presence of chronic villitis of unknown etiology. Treatment with acetylsalicylic acid, heparin, steroids, and hydroxychloroquine has been reported with mixed outcomes [15,16].

Chronic intervillositis of unknown etiology or chronic histiocytic intervillositis (CIUE/CHI) is the description of a microscopic appearance of an inflammatory placental disease in which maternal histiocytes fill the intervillous space. The non-specific chronic histiocytic intervillositis has been firstly described in the literature by Labarrere and Mullen in 1987 [37]. Chronic intervillositis of unknown etiology lesions are diffuse of multifocal, located in the intervillous space and including extensive infiltration of inflammatory cells, mainly mononuclear cells of maternal origin, fibrin deposits, and trophoblastic erosion of varying degrees [30]. Although it is a rare disease, chronic intervillositis of unknown etiology is an important cause of concern as it is linked to severe obstetric complications such as early and late spontaneous abortions, intrauterine growth restriction, and in utero deaths. The typical appearance of the mononuclear cells that infiltrate the intervillous space are CD68 and CD45, suggesting a potential immunologic cause, although no specific antigens have been associated [30].

## 3. Pregnancy Complications Associated with Chronic Placental Inflammation

**Figure 2:**
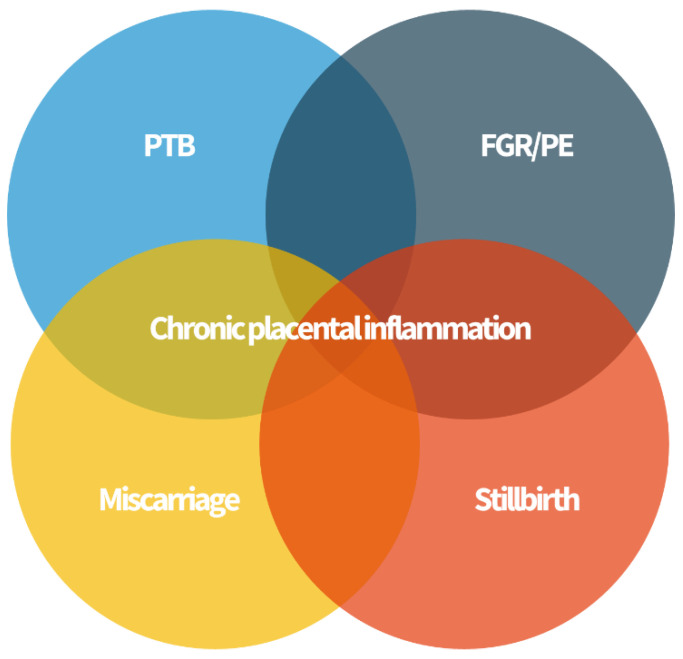
Chronic placenta inflammation is found in some cases of preterm birth (PTB), fetal growth restriction (FGR) and preeclampsia (PE), miscarriage, and stillbirth. These “great obstetrical syndromes” may share a common pathophysiology.

All forms of chronic placental inflammation have possible associations with poor obstetric outcomes [34]. Complications such as PTB, FGR and neurocognitive and development disorders, recurrent miscarriage, stillbirth, and neonatal alloimmune thrombocytopenia (NAIT) have been associated with inflammation of the placenta of unknown etiology after a careful histopathological analysis (Figure 2).

Chronic villitis of unknown etiology and chronic deciduitis with plasma cells have been associated with preterm labor, but the inflammatory process has not been considered as an independent risk factor for long-term outcomes [34]. Acute chorioamnionitis remains the most common cause associated with early PTB considered less than 28 weeks, and chronic chorioamnionitis is most frequently associated with late preterm birth between 34 and 37 weeks [38].

In terms of FGR, CVUE have been associated with poor fetal growth, low birth weight, and small for gestational age fetuses with rates of 70% [32]. Follow-up in children with FGR from pregnancies with histological expression of CVUE demonstrated an increased risk of low developmental index at 2 years of age and increased risk of cerebral palsy and abnormal neurodevelopmental findings in term infants [39]. Injury in the basal ganglia and thalamus has been described in a study with term infants with hypoxic ischemic encephalopathy and chronic villitis at the level of the placenta [40].

A rare association is neonatal alloimmune thrombocytopenia (NAIT) and CVUE. A histopathological study that histopathological examined 14 placentas of pregnancies affected by NAIT showed CVUE as primary lesions [41]. Neonatal alloimmune thrombocytopenia is a rare pregnancy complication characterized by a destruction of platelets in the fetus or infant due to a mismatch between the mother’s platelets and those of the baby. As NAIT is characterized by alloimmunization, the association with CVUE provides further evidence that the process may be immune related.

Sadly, many cases of histological diagnosis of chronic placental inflammation were associated with stillbirth and recurrent pregnancy loss [34]. Chronic intervillositis of unknown etiology (CIUE) is strongly associated with miscarriage, intrauterine fetal demise, and a very high risk of recurrence [42].

In pregnancies complicated by the “great obstetrical syndromes” the placenta should always be sent for examination to a specialized pathologist (placenta pathologist or perinatal pathologist).

## 4. Results and Discussion: Hydroxychloroquine in Pregnancy

### 4.1. HCQ Used in Pregnancy to Improve Outcomes in Women with Autoimmune Conditions

Hydroxychloroquine is an antimalarial agent firstly described in 1955 that has an anti-inflammatory and immunomodulator effect, showing a complex mechanism of action. It increases intracellular pH within intracellular vacuoles, and it has been demonstrated that it alters various processes such as protein degradation by acidic hydrolases in the lysosome, assembly of macromolecules in the endosomes, and post-translation modification of proteins in the Golgi apparatus [43]. It has also been suggested that HCQ is involved in phagocytosis, proteolysis, antigen presentation, and chemotaxis, decreasing the production of pro-inflammatory cytokines and prostaglandins, inhibiting of matrix metalloproteinases, and blocking T- and B-cell receptor and toll-like receptor signaling [44]. Additionally, HCQ has lipid lowering, anticoagulant, and antidiabetic effects that may take part in reducing high cardiovascular risk in autoimmune diseases [45]. Regarding possible effects on trophoblastic placental tissue, recent studies have shown HCQ to partially reverse antiphospholipid antibody-induced inhibition of trophoblast migration and to restore the diminished trophoblast fusion and function [46].

Hydroxychloroquine is prescribed for inflammatory conditions associated with adverse perinatal outcome (Table 2) such as systemic lupus erythematosus (SLE), antiphospholipid syndrome (APLS), and placental inflammatory lesions such as CIU/CHI [47]. Table 2 summarizes the studies that have reported use of HCQ during pregnancy with improvement of pregnancy outcomes.

With regards to safety of HCQ in pregnancy, studies from the literature have suggested no hydroxychloroquine-related adverse effects on the fetus [20,50,61,62,63,64,65], with the exception of one meta-analysis that showed an increased rate of spontaneous pregnancy when HCQ was administered in the first weeks of pregnancy [66]. However, underlying conditions were not excluded when doing the meta-analysis, and therefore the result was potentially biased and thus further research is needed to provide accurate information. Hydroxychloroquine has a good oral absorption, long plasma elimination, and slow renal clearance and crosses the placental barrier, being found at similar concentrations in both the umbilical cord blood and maternal blood. Several studies found that there was no clinical ocular toxicity in pregnancy and that it has good risk-to-benefit balance in treating immunological impairment and chronic endothelial dysfunction of pregnant women [67]. Given the safety profile of HCQ in pregnancy, in the last years, the use during pregnancy complicated by autoimmune disorders has increased significantly.

A meta-analysis regarding safety administration of HCQ reported no increase in the risk of major congenital malformations, spontaneous abortions, fetal death, and prematurity [68]. Another systematic review suggested a possible protective effect of HCQ use on the occurrence and recurrence of cardiac neonatal lupus and congenital heart block [66]. Hydroxychloroquine may regulate the autoimmune-mediated inflammation of the fetal heart in fetuses at risk of atrio-ventricular block from mothers with anti-Ro/SSA and anti-La-/SSB antibodies by reducing the level of maternal antibodies and translocating through the placenta, thereby preventing the immune damage of the fetal heart [69,70].

Hydroxychloroquine exerts a strong and persistent anti-inflammatory response at the level of trophoblastic tissue, as previously studied in excessive inflammation that causes placental insufficiency in antiphospholipid syndrome-complicated pregnancies [71]. Antiphospholipid syndrome is described by circulating antiphospholipid antibodies that determine an excessive inflammatory response and increase the chance of adverse pregnancy complication, such as recurrent pregnancy loss, preeclampsia, HELLP syndrome (hemolytic anemia, elevated liver enzymes, and low platelet counts), intrauterine growth restriction, and premature delivery. Albert et al. [71] showed that hydroxychloroquine partially antagonizes the inhibition of trophoblast migration from antiphospholipid syndrome, possibly through the modulation of IL-6 production, and may prevent Toll-like receptor 4-dependent trophoblast inflammatory cytokine response.

### 4.2. Hydroxychloroquine in Chronic Histiocytic Intervillositis (CHI)

A study from the Children’s and Women’s Hospital in Vancouver, BC, Canada [72], reports various empiric treatment in patients with previous diagnosis of CIUE/CHI. One of the options of treatment was HCQ with a dosage ranging between 200 and 400 mg PO daily. It was started pre-pregnancy or with a positive pregnancy test and continued until pregnancy loss or delivery. None of the cases of pregnant women treated with HCHQ developed retinopathy, a rare complication from hydroxychloroquine use. In patients with CIUE/CHI, serum alkaline phosphatase was increased 2.5 times as compared to normal values, and it may be a potential marker that can be followed in subsequent pregnancies [73].

Another team studied in vitro the effect of HCQ on restoring trophoblastic cell fusion and differentiation by anti-β2GP1 antibodies in anti-phospholipidic syndrome after it was already demonstrated that HCQ has been used for the prevention on fetal losses in patients with SLE by restoring the annexin V shield, a trophoblastic anticoagulant protection on the cell surface. The plasma of patients with anti-phospholipid syndrome and the anti- β2GP1 antibodies severely decrease trophoblastic cell fusion and differentiation. This new mechanism in fetal loss can be interfered by HCQ by inhibiting toll-like receptor 4 and to reduce the effect of previously mentioned antibodies [74].

Scott et al. [75] reviewed the effects of HCHQ on placental function, structure, and nutrient transport in term villous explants in order to investigate whether HCHQ can modulate placental cytokine secretion in response to activation of toll-like receptor 7 and 9. Hydroxychloroquine had potentially beneficial effects by significantly increasing the secretion of IL-10, an anti-inflammatory cytokine that plays an important role in the placenta inducing maternal tolerance of the allogeneic fetus. Thus, the relationship between HCHQ upregulation of IL-10 and the increased rates of live births are seen following hydroxychloroquine in women with antiphospholipid syndrome [23].

It has been observed that beside increasing the release of IL-10, the treatment with HCQ (1.5 µg/mL) increased the number of villi with intact syncytiotrophoblast at day 7 of explant culture and reduced the number of villi with vacuolated stroma. Improving the regeneration of the critical cell layer and smaller number of vacuoles are associated with better transplacental transport and exchange [75]. The therapeutic potential of hydroxychloroquine was assessed in relation to the modulatory response to activation of toll-like receptor 7 and 9 that are overstimulated in preeclampsia, antiphospholipidic syndrome, or inflammatory placental lesions [76].

Mekinian et al. [47] demonstrated in a multicenter prospective study outcomes associated with maternal disease and treatment in cases with known CIU/CHI. They included 24 cases and described the associated autoimmune maternal conditions, pregnancy outcomes, and treatment. CHI was found to be frequently associated with other autoimmune conditions. Hydroxychloroquine was used in the treatment of six cases in combination with aspirin, prednisolone, and low molecular heparin. The number of women treated with HCQ was too small to formulate a conclusion about HCQ in this study; however, in treated pregnancies, there was a trend on improving pregnancy outcomes [47].

In a retrospective study of pregnant women with at least one pregnancy complicated with CHI, the team of Brady et al. [27] showed that the use of hydroxychloroquine alone or in combination with prednisolone in the treatment of CHI resulted in a reduction of disease severity and a trend toward an increase in livebirth rate in women with known CHI [27].

Less has been studied about the possible utility of HCQ in preventing recurrent chronic villitis of unknown etiology.

## 5. Conclusions

The demonstration of the beneficial effects of HCQ to prevent maternal and fetal complications has recently opened the way for its use for inflammatory disorders of the placenta with the aim of improving pregnancy outcomes [77,78].

Chronic inflammatory lesions of the placenta are rare but associated with severe obstetrical outcomes, and they tend to recur in subsequent pregnancies. Several therapeutic interventions have been used to prevent recurrence of placental inflammation, in particular CHI/CIUE in subsequent pregnancies. There is the potential for HCQ to be useful in preventing CPI lesions, as suggested by in vitro research, as well as recently by prospective and retrospective human studies. Further properly designed, probably multicentric (because of the rarity of CPI of unknown etiology) studies are needed to determine the utility, safety, and mechanism of action of HCQ in placental tissue disease. A uniform classification with broad acceptance and clinical use of the histological aspect of chronic placental lesions is required beforehand. Moreover, increased awareness by physicians about the possible involvement of chronic placental inflammation, often linked to maternal immune alterations, in adverse pregnancy outcomes is important.

## Figures and Tables

**Table 1:** Chronic placental inflammation—classification.

Chronic placenta inflammation associated with specific maternal infections (COVID-19, cytomegalovirus, Treponema pallidum, HIV, Zika).
Chronic placental inflammation of unknown etiology - eosinophilic/T-cell vasculitis; - chronic villitis (CVUE); - chronic intervillositis of unknown origin, chronic histiocytic intervillositis (CIUE, CHI); - chronic deciduitis.

**Table 2:** Hydroxychloroquine and autoimmune disorders in pregnancy.

Author and Year	Type of Study	Indication
Buchanan et al., (1996) [48]	Observational cohort	Systemic/discoid lupus erythematosus
Levy et al., (2001) [49]	Randomized control	Systemic/discoid lupus erythematosus
Costedoat-Chalumeau et al., (2003) [50]	Observational cohort	Systemic lupus erythematosus, Sjögren syndrome
Frassi et al., (2004) [51]	Observational cohort	Connective tissue disease
Clowse et al., (2006) [52]	Observational cohort	Systemic lupus erythematosus
Diav-Citrin et al., (2013) [53]	Observational cohort	Systemic lupus erythematosus, rheumatic arthritis, Behcet’s disease, Sjögren syndrome
Cooper et al., (2014) [54]	Observational cohort	Rheumatoid arthritis, psoriatic arthritis, ankylosing spondylitis, systemic lupus erythematosus, scleroderma, inflammatory bowel disease
Gayed et al., (2014) [55]	Observational cohort	Systemic lupus erythematosus
Erkan et al., (2017) [56]	Observational cohort	Systemic lupus erythematosus
Izmirly P. et al., (2020) [26]	Randomized control	Prevention of fetal atrioventricular block for the fetus
Gerde M. et al., (2021) [57]	Observational cohort	Refractory obstetric antiphospholipid syndrome
Duan J. et al., (2021) [58]	Meta-analysis	Prophylaxis of preeclampsia, hypertension, and prematurity in pregnancies with SLE
Berard A. et al., (2012) [19]	Observational cohort	Treatment of SLE and rheumatoid arthritis
Beksac M.S. et al., (2021) [21]	Observational cohort	Autoimmune disorders, autoimmune antibody positivities, and chronic inflammatory/autoimmune diseases
Pasquier E. et al., (2019) [59]	Randomized placebo-controlled trial	Prevention of recurrent miscarriage
de Moreuil C. et al., (2020) [60]	Review of literature	Potential benefit in preeclampsia and recurrent miscarriage

## References

[1] Thornburg K.L., Marshall N. (2015). The placenta is the center of the chronic disease universe. Am. J. Obstet. Gynecol..

[2] Jiang B., Godfrey K.M., Martyn C.N., Gale C.R. (2006). Birth weight and cardiac structure in children. Pediatrics.

[3] Martyn C., Greenwald S. (2001). A hypothesis about a mechanism for the programming of blood pressure and vascular disease in early life. Clin. Exp. Pharmacol. Physiol..

[4] Luyckx V., Brenner B. (2015). Birth weight, malnutrition and kidney-associated outcomes—A global concern. Nat. Rev. Nephrol..

[5] Dumortier O., Blondeau B., Duvillie B., Reusens B., Breant B., Remacle C. (2007). Different mechanisms operating during different critical time-windows reduce rat fetal beta cell mass due to maternal low-protein or low-energy diet. Daibetologia.

[6] Buss C., Entringer S., Wadhwa P.D. (2012). Fetal programming of brain development: Intrauterine stress and susceptibility to psychopathology. Sci. Signal.

[7] Roberts D.J., Celi A.C., Riley L.E., Onderdonk A.B., Boyd T.K., Johnson L.C., Lieberman E. (2012). Acute histologic chorioamnionitis at term: Nearly always noninfectious. PLoS ONE.

[8] Khong T.Y., Mooney E.E., Ariel I., Balmus N.C., Boyd T.K., Brundler M.A., Derricott H., Evans M.J., Faye-Petersen O.M., Gillan J.E. (2016). Sampling and Definitions of Placental Lesions:Amsterdam Placental Workshop Group Consensus Statement. Arch. Pathol. Lab. Med..

[9] Romero R., Miranda J., Kusanovic J.P., Chaiworapongsa T., Chaemsaithong P., Martinez A., Gotsch F., Dong Z., Ahmed A.I., Shaman M. (2015). Clinical chorioamnionitis at term I: Microbiology of the amniotic cavity using cultivation and molecular techniques. J. Perinat. Med..

[10] Ovalle A., Martínez M.A., Kakarieka E., Gómez R., Torres J., Fuentes A., Ruiz M., Angel R. (1998). Placental histopathology in premature rupture of membranes. Its relationship with microbiological findings, maternal and neonatal outcome. Rev. Med. Chil..

[11] Romero R., Espinoza J., Gonçalves L.F., Kusanovic J.P., Friel L.A., Nien J.K. (2006). Inflammation in preterm and term labour and delivery. Semin. Fetal Neonatal Med..

[12] Challier J.C., Basu S., Bintein T., Minium J., Hotmire K., Catalano P.M., Hauguel-de Mouzon S. (2008). Obesity in pregnancy stimulates macrophage accumulation and inflammation in the placenta. Placenta.

[13] Labarrere C.A., Hardin J.W., Haas D.M., Kassab G.S. (2015). Chronic villitis of unknown etiology and massive chronic intervillositis have similar immune cell composition. Placenta.

[14] Redline R. (2007). Villitis of unknown etiology: Noninfectious chronic villitis in the placenta. Hum. Pathol..

[15] Contro E., DeSouza R., Bhide A. (2010). Chronic intervillositis of the placenta: A systematic review. Placenta.

[16] Vardi L., Paterson H., Hung N.A. (2017). Successful pregnancy following treatment of recurrent chronic histiocytic intervillositis. BMJ Case Rep..

[17] Boog G., Le Vaillant C., Alnoukari F., Jossic F., Barrier J., Muller J. (2006). Combining corticosteroid and aspirin for the prevention of recurrent villitis or intervillositis of unknown etiology. J. Gynecol. Obstet. Biol. Reprod..

[18] Abdulghani S., Moretti F., Gruslin A., Grynspan D. (2017). Recurrent massive perivillous fibrin deosition and chronic intervillositis treated with heparin and intravenous immunoglobulin: A case report. J. Obstet. Gynecol. Can..

[19] Bérard A., Sheehy O., Zhao J.P., Vinet E., Quach C., Bernatsky S. (2012). Chloroquine and Hydroxuchloroquine use during pregnancy and the risk of adverse pregnancy outcomes using real-world evidence. Front Pharmacol..

[20] Chambers C.D., Johnson D.L., Xu R., Luo Y., Felix R., Fine M., Lessard C., Adam M.P., Braddock S.R., Robinson L.K. (2021). Birth outcomes in women who have taken hydroxycholoroquine in pregnancy: A prospective cohort study. Arthritis Rheumatol..

[21] Beksac M.S., Donmez H.G. (2021). Impact of hydroxychloroquine on the gestational outcomes of pregnant women with immune system problems that necessitate the use of the drug. J. Obstet. Gynecol. Res..

[22] Canti V., Scarrone M., De Lorenzo R., Ramirez G.A., Erra R., Bordoli S., Cella S., Schmit E., Rosa S., Castiglioni M.T. (2021). Low incidence of intrauterine growth restriction in pregnant patients with systemic lupus erythematosus taking hydroxychloroquine. Immunol. Med..

[23] Sciascia S., Branch D.W., Levy R.A., Middeldorp S., Pavord S., Roccatello D., Ruiz-Irastorza G., Tincani A., Khamashta M., Schreiber K. (2016). The efficacy of hydroxychloroquine in altering pregnancy outcome in women with antiphospholipid antibodies. Evidence and clinical judgment. Thromb. Haemost..

[24] Koh J.H., Ko H.S., Kwok S.K., Ju J.H., Park S.H. (2015). Hydroxychloroquine and pregnancy on lupus flares in Korena patients with systemic lupus erythematosus. Lupus.

[25] Luo Y., Zhang L., Fei Y., Li Y., Hao D., Liu Y., Zhao Y. (2015). Pregnancy outcome of 126 andti-SSA/Ro-positive patients during the past chloroquine and hydroxychloroquine Infectious, Immune, Neoplastic and Neurological disease 24 years-a retrospective cohort study. Clin. Rheumatol..

[26] Izmirly P., Kim M., Friedman D.M., Costedoat-Chalumeau N., Clancy R., Copel J.A., Phoon C.K., Cuneo B.F., Cohen R.E., Robins K. (2020). Hydroxychloroquine to prevent recurrent congenital heart block in fetuses of anti-SSA/Ro-positive mothers. J. Am. Coll. Cardiol..

[27] Brady C.A., Williams C., Batra G., Church E., Tower C.L., Crocker I.P., Heazell A.E. (2021). Immunomodulatory Therapy Reduces the Severity of Placental Lesions in Chronic Histiocyti Intervillositis. Front. Med..

[28] Pearson H. (2021). How COVID broke the evidence pipeline. Nature.

[29] Katzman P., Oble D. (2013). Eosinophilic/T-cell chronic vasculitis and chronic villitis involve regulatory T cells and often occur together. Pediatr. Dev. Pathol..

[30] Marchaudon V., Devisme L., Petit S., Ansart-Franquet H., Vaast P., Subtil D. (2011). Chronic histiocytic intervillositis of unknown etiology: Clinical features in a consecutive series of 69 cases. Placenta.

[31] Boyd T., Redline R. (2000). Chronic histiocytic intervillositis: A placental lesion associated with recurrent reproductive loss. Hum. Pathol..

[32] Nowak C., Joubert M., Jossic F., Masseau A., Hamidou M., Philippe H.J., Le Vaillant C. (2016). Perinatal prognosis of pregnancies complicated by placental chronic villitis or intervillositis of unknown etiology and combined lesions: About a series of 178 cases. Placenta.

[33] Bos M., Nikkels P.G., Cohen D., Schoones J.W., Bloemenkamp K.W., Bruijn J.A., Baelde H.J., van der Hoorn M.L.P., Turner R.J. (2018). Towards standardized criteria for diagnosing chronic intervillositis of unknown etiology. A systematic review. Placenta.

[34] Kim C.J., Romero R., Chaemsaithong P., Kim J.S. (2015). Chronic inflammation of the placenta: Definition, classification, pathogenesis and clinical significance. Am. J. Obstet. Gynecol..

[35] Raman K., Wang H., Troncone M.J., Khan W.I., Pare G., Terry J. (2015). Overlap chronic placental inflammation is associated with a unique gene expression pattern. PLoS ONE.

[36] Sato Y. (2021). Inflammatory lesions in placental pathology. J. Obstet. Gynecol. Res..

[37] Labarrere C., Mullen E. (1987). Fibrinoid and Trophoblastic Necrosis with Massive Chronic Intervillositis: An Extreme Variant of Villitis of Unknown Etiology. Am. J. Reprod. Immunol..

[38] Lee J., Kim J.S., Park J.W., Park C.W., Park J.S., Jun J.K., Yoon B.H. (2013). Chronic chorioamnionitis is the most common placental lesion in late preterm birth. Placenta.

[39] Torrance H.L., Bloemen M.C.T., Mulder E.J.H., Nikkels P.G.J., Derks J.B., De Vries L.S., Visser G.H.A. (2010). Predictors of outcome at 2 years of age after early intrauterine growth restriction. Ultrasound Obstet. Gynecol..

[40] Harteman J.C., Nikkels P.G., Benders M.J., Kwee A., Groenendaal F., de Vries L.S. (2013). Placental pathology in full-term infants with hypoxic-ischemic neonatal encephalopathy and association with magnetic resonance imaging pattern of brain injury. J. Pediatr..

[41] Althaus J., Weir E.G., Askin F., Kickler T.S., Blakemore K. (2005). Chronic villitis in untreated neonatal alloimmune thrombocytopenia: An etiology for severe early intrauterine growth restriction and the effect of intravenous immunoglobulin therapy. Am. J. Obstet. Gynecol..

[42] Derricott H., Jones R.L., Greenwood S.L., Batra G., Evans M.J., Heazell A.E. (2016). Characterizing Villitis of Unknown Etiology and Inflammation in Stillbirth. Am. J. Pathol..

[43] Fox R. (1993). Mechanism of action of hydroxychloroquine as an atirheumatic drug. Semin. Arthritis Rheum..

[44] Costedoat-Chalumeau N., Dunogué B., Morel N., Le Guern V., Guettrot-Imbert G. (2014). Hydroxychloroquine: A multifaceted treatment in lupus. Presse Med..

[45] Belizna C. (2015). Hydroxuchloroquine as an anti-thrombotic in antiphodpholipid syndrome. Autoimmun. Rev..

[46] Dong Y., Lu Y., Xia Y., Wang X. (2021). Effect of hydroxychloroquine on antiphospholipid antibodies-inhibited endometrial angiogenesis. J. Matern.-Fetal Neonatal Med..

[47] Mekinian A., Costedoat-Chalumeau N., Masseau A., Botta A., Chudzinski A., Theulin A., Emmanuelli V., Hachulla E., De Carolis S., Revaux A. (2015). Chronic histiocytic intervillositis: Outcome, associated disease and treatment in a multicenter prospective study. Autoimunity.

[48] Buchanan N.M., Toubi E., Khamashta M.A., Lima F., Kerslake S., Hughes G.R. (1996). Hydroxychloroquine and lupus pregnancy: Review of a series of 36 cases. Ann. Rheum. Dis..

[49] Levy R.A., Vilela V.S., Cataldo M.J., Ramos R.C., Duarte J.L., Tura B.R., Albuquerque E.M., Jesus N.R. (2001). Hydroxychloroquine in lupus pregnancy: Double-blind and placebo-controlled study. Lupus.

[50] Costedoat-Chalumeau N., Amoura Z., Lechat P., Piette J.C. (2005). Safety of hydroxychloroquine in pregnant patients with connective tissue diseases. Review of the literature. Autoimmun. Rev..

[51] Frassi M., Biasini C., Taglieti M., Daneli E., Faden D., Lojacono A., Motta M., Gerosa M., Meroni P.L., Doria A. (2004). Hydroxychloroquine in pregnant patients with rheumatic disease: A case control observation of 76 treated pregnancies. Lupus.

[52] Clowse M.E., Magder L., Witter F., Petri M. (2006). Hydroxychloroquine in lupus pregnancy. Arthritis Rheum..

[53] Diav-Citrin O., Blyakhman S., Shechtman S., Ornoy A. (2013). Pregnancy outcome following in utero exposure to hydroxychloroquine: A prospective comparative observational study. Reprod. Toxicol..

[54] Cooper W.O., Cheetham T.C., Li D.K., Stein C.M., Callahan S.T., Morgan T.M., Shintani A.K., Chen N., Griffin M.R., Ray W.A. (2014). Risk of adverse fetal outcomes associated with immunosuppressive medications for chronic immune-mediated disease in pregnancy. Arthritis Rheum..

[55] Gayed M., Khamashta M., Culliford D., Leone F., Toescu V., Bruce I., Giles I., Teh L.S., Mc Hugh N., Akil M. (2014). Longterm outcomes of children born to mothers with SLE exposed to hydroxychloroquine in pregnancy. Rheumatology.

[56] Erkan D., Unlu O., Sciascia S., Belmont H.M., Branch D.W., Cuadrado M.J., Gonzalez E., Knight J.S., Uthman I., Willis R. (2017). Hydroxychloroquine in the primary thrombosis prophylaxis of antiphospholipid antibody positive patients without systemic autoimmune disease. Lupus.

[57] Gerde M., Ibarra E., Mac Kenzie R., Suarez C.F., Heer C., Alvarez R., Iglesias M., Balparda J., Beruti E., Rubinstein F. (2021). The impact of hydroxychloroquine on obstetric outcomes in refractory obstetric antiphospholipid syndrome. Thromb. Res..

[58] Duan J., Ma D., Wen X., Guo Q., Gao J., Zhang G., Xu K., Zhang L. (2021). Hydroxychloroquine prophylaxis for preeclampsia, hypertension and prematurity in pregnant patients with systemic lupus erythematosus: A meta-analysis. Lupus.

[59] Pasquier E., de Saint-Martin L., Marhic G., Chauleur C., Bohec C., Bretelle F., Lejeune-Saada V., Hannigsberg J., Plu-Bureau G., Cogulet V. (2019). Hydroxychloroquine for prevention of recurrent miscarriage: Study protocol for a multicentre randomised placebo-controlled trial BBG study. BMJ Open.

[60] de Moreuil C., Alavi Z., Pasquier E. (2020). Hydroxychloroquine may be beneficial in preeclampsia and recurrent miscarriage. Br. J. Clin. Pharmacol..

[61] Howley M.M., Werler M.M., Fisher S.C., Van Zutphen A.R., Carmichael S.L., Broussard C.S., Heinke D., Ailes E.C., Pruitt S.M., Reefhuis J. (2021). Maternal exposure to hydroxychloroquine and birth defects. National Birth Defects Prevention Study. Birth Defects Res..

[62] Huybrechts K.F., Bateman B.T., Zhu Y., Straub L., Mogun H., Kim S.C., Desai R.J., Hernandez-Diaz S. (2021). Hydroxychloroquine early in pregnancy and risk of birth defects. Am. J. Obstet. Gynecol..

[63] Paizis K. (2019). Immunomodulatory drugs in pregnancy and lactation. Aust. Prescr..

[64] Lacroix I., Bénévent J., Damase-Michel C. (2020). Chloroquine and hydroxychloroquine during pregnancy: What do we know?. Therapie.

[65] Birru Talabi M., Clowse M. (2020). Antirheumatic medications in pregnancy and breastfeeding. Curr. Opin. Rheumatol..

[66] Kaplan Y.C., Ozsarfati J., Nickel C., Koren G. (2016). Reproductive outcomes following hydroxychloroquine use for autoimune disease: A systematic review and meta-analysis. Br. J. Clin. Pharmacol..

[67] Zhan Z., Yang Y., Zhan Y., Chen D., Liang L., Yang X. (2017). Fetal outcomes and associated factors of adverse outcomes of pregnancyin southern Chinese women with systemic lupus erythematosus. PLoS ONE.

[68] Sperber K., Hom C., Chao C.P., Shapiro D., Ash J. (2009). Systematic review of hydroxychloroquine use in pregnant patients with autoimune disease. Pediatr. Rheumatol. Online J..

[69] Zhou K., Zhao L., Zhou Y., Wang C., Li Y., Zhu Q., Hua Y., Qiao L. (2021). Successful prevention of fetal autoimmune-mediated heart block by combined therapies with hydroxychloroquine aand intravenous immunoglubulin: A case report. Front. Cardiovasc. Med..

[70] Ciobanu A.M., Dumitru A.E., Gica N., Botezatu R., Peltecu G., Panaitescu A.M. (2020). Benefits and risks of IgG Tranplacental Transfer. Diagnostics.

[71] Albert C.R., Schlesinger W.J., Viall C.A., Mulla M.J., Brosens J.J., Chamley L.W., Abrahams V.M. (2014). Effect of Hydroxycholoquine on Antiphospholipid Antibody-Induced Changes in First Trimester Throphoblast Function. Am. J. Reproductiv. Immun..

[72] Simula N.K., Terry J., Kent N.E., Robertson J., Purkiss S., Bloomenthal D., Williams C., Bedaiwy M.A. (2020). Chronic Intervillositis of Unknown Etiology: Prevalence, patterns and reproductive outcomes at a tertiary referral institution. Placenta.

[73] Koby L., Keating S., Malinowski A.K., D’Souza R. (2018). Chronic histiocytic intervillositis—clinical, biochimical and radiological findings: An observational study. Placenta.

[74] Marchetti T., Ruffatti A., Wuillemin C., De Moerloose P., Cohen M. (2014). Hydroxychloroquine restores trophoblast fusion affected by antiphospholipid antibodies. J. Thromb. Haemost..

[75] Scott R.E., Greenwood S.L., Hayes D.J., Baker B.C., Jones R.L., Heazell A.E. (2019). Effects of Hydroxychloroquine on the Human Placenta-Findings from In Vitro Experimental Data and a Systematic Review. Reprod. Toxicol..

[76] Hussein K., Stucki-Koch A., Kreipe H., Feist H. (2015). Expression of Toll-Like Receptors in Chronic Histiocytic Invervillositis of the Placenta. Fetal. Pediatr. Pathol..

[77] Brook A., Francis K., Hansson S.R., Crocker I.P., Brownbill P. (2017). Hydroxychloroquine ameliorates FGR-associated free fetal haemoglobin evoked fetoplacental vasoconstriction. Placenta.

[78] Kim M.J., Romero R., Kim C.J., Tarca A.L., Chhauy S., LaJeunesse C., Lee D.C., Draghici S., Gotsch F., Kusanovic J.P. (2009). Villitis of unknown etiology is associated with a distinct pattern of chemokine up-regulation in the feto-maternal and placental compartimets: Implication of conjoint maternal allograft rejection and maternal anti-fetal graft-versus host disease. J. Immunol..

